# A Study to Improve the Vitamin A and Iodine Status of Pregnant Women through a Multiple Micronutrient Fortified Salt

**DOI:** 10.1155/2022/5301499

**Published:** 2022-11-10

**Authors:** Malavika Vinod Kumar, M. Sankarapandian, Juergen Erhardt

**Affiliations:** ^1^Sundar Serendipity Foundation, 6G Century Plaza, 560/562 Anna Salai, Chennai 600018, India; ^2^The Micronutrient Research Foundation, 6G Century Plaza, 560/562 Anna Salai, Chennai 600018, India; ^3^The Gandhigram Institute of Rural Health and Family Welfare Trust, Soundram Nagar, Gandhigram Post, Dindigul 624302, India; ^4^VitMin Lab, Kastanienweg 5, Willstaett 77731, Germany

## Abstract

**Background:**

To study the efficacy of a multiple micronutrient fortified salt enriched with iron, iodine, vitamin A, vitamin B1, vitamin B2, vitamin B6, vitamin B12, niacin, and folic acid in improving serum retinol and iodine status of pregnant women.

**Methods:**

It was a randomized control trial in the antenatal clinic of a hospital. 151 women in the experimental group received a multiple micronutrient fortified salt to cook all their meals, and 150 women in the control group did not receive the fortified salt. Blood was collected in the three trimesters. Urine was collected in their first and third trimesters. Serum retinol, CRP (C-reactive protein), and AGP (Alpha glycoprotein) in blood were assessed, and iodine was assessed in urine. All the women were dewormed once.

**Results:**

The inflammation adjusted mean serum retinol in three trimesters in the experimental group was 24.51, 27.29, and 25.68 *µ*g/dL, and it was 28.97, 27.63, and 22.72 *µ*g/dL in the control group. Over the study period of 6 months, the increase in serum retinol in the experimental group was 1.17 *µ*g/dL whereas in the control group serum retinol decreased by 6.25 *µ*g/dL. The experimental group increase in serum retinol is significantly more (*p*=0.0001) than the changes in retinol in the control group. The prevalence of serum retinol deficiency in the three trimesters was 39.1%,25.8%, and 37.7% in the experimental group and 14%, 22.7%, and 39.3% in the control group, and the change in the experimental group was significant (*p*=0.001) compared to the control group by binary logistic regression. Over the study period of 6 months there is a significant increase in urinary iodine concentration in the experimental group (*p*=0.030), showing absorption of iodine from the fortified salt whereas there is a significant decline in the iodine values in the control group (*p*=0.008). At the end of the study, the urinary iodine concentration of the experimental group was significantly more (*p*=0.0001) than that of the control group.

**Conclusion:**

The fortified salt was able to improve serum retinol levels and urinary iodine levels in pregnant women. *Trial Registration*. This trial was registered retrospectively on 19/02/2022 in the ISRCTN registry with trial ID ISRCTN17782574.

## 1. Background

Multiple micronutrient deficiencies are prevalent in many developing countries including India [1]. Women and children are affected predominantly. It is well recognized globally that nutrition during pregnancy is critical for the well-being of the offspring, and micronutrient deficiencies are common in low and middle income countries (LMIC), including India. Physiologically, it is also known that pregnancy is the period when requirements of several micronutrients increase, and diets in vulnerable target population like pregnancy is far from meeting the needs. Anaemia is mentioned as a common nutritional problem in men and women, whereas there is no large scale survey on vitamin A deficiency.

The National Family Health Survey (NFHS5) done by the Government of India in 2019–2020 shows that in Tamilnadu, 48.3% of pregnant women are anaemic [2]. Though the NFHS surveys tests for anaemia are done, tests for serum vitamin A are not done. In our previous studies in nearby areas, we have seen that vitamin A deficiency is prevalent in about 50% of children [3]. We therefore decided to improve the vitamin A and iodine status of pregnant women attending the antenatal clinic of a hospital by using a multiple micronutrient fortified salt in this study. Iodine is a mandatory micronutrient in salt fortification in India, and it was one of the micronutrients in the fortified salt in this study as well. Iodine is a very important micronutrient for the proper growth and development of the fetus. Therefore in this study, urinary iodine was tested in the pregnant women in the first trimester and the third trimester.

### 1.1. Aim

To improve serum retinol (vitamin A) and urinary iodine levels in pregnant women through the use of a multiple micronutrient fortified salt, enriched with iron, iodine, vitamin A, vitamin B1, B2, B6, B12, niacin, and folic acid, in cooking all the meals.

## 2. Methods

### 2.1. Study Design

The study was a randomized controlled trial to study the efficacy of the multiple micronutrient fortified salt in improving serum retinol and urinary iodine values in the pregnant women to improving their vitamin A and iodine status.

### 2.2. Sample Size

The sample size was calculated using the formula:

(1) $$\begin{array}{c} P\left(1-P\right){\left(\frac{1.96}{E}\right)}^{2}, \end{array}$$

where P stands for the current proportion of women having serum vitamin A deficiency, and *E* is the difference in the proportion of women having serum vitamin A deficiency which will be brought down because of the intervention. In a pilot study done in the hospital, it was seen that the prevalence of serum vitamin A deficiency was about 30% in the women. If we assume that the reduction of vitamin A deficiency will be from 30% to 22% because of the intervention, the sample size is

(2) $$\begin{array}{c} \text{0}.3\left(1-\text{0}.3\right){\left(\frac{1.96}{\text{0}.08}\right)}^{2}=126, \end{array}$$

where *P*=0.3 because the prevalence is 30%. *E* = 0.08. (0.3–0.22 = 0.08).

Giving margin for dropouts, it was decided that the sample size would be 150 women in each group. We arrived at the sample size for urinary iodine based on our previous studies. We have considered an *α* of 0.05 and power of 0.80 and a two tailed test and arrived at a sample size of 100 in each group for urinary iodine.

### 2.3. Study Location

Kasturba hospital, Gandhigram, Dindugal district, South India is the location of study. The pregnant women who attended the antenatal clinic of this hospital were enrolled into the study.

### 2.4. Inclusion Criteria

Pregnant women, in the age group 19 years to 29 years, in the first trimester of pregnancy who gave written informed consent were included in the study.

### 2.5. Exclusion Criteria

Women with hemoglobin less than 8 grams/dL were excluded so that they could be treated immediately for anaemia. Women with current or previous gynecological problems were also excluded.

### 2.6. Experimental and Control Groups

The women who visited the antenatal clinic were included in the study and they were placed into the experimental or control group based on the week they visited the antenatal clinic. The women would be enrolled into the experimental group in one week, and the women who visited the clinic next week would be enrolled into the control group. This process continued until the required numbers of women were enrolled in the study. The women were included in the study only if they were in the first trimester of their pregnancy, based on the date of their last menstrual period.

### 2.7. Intervention

The women in the experimental group received a multiple micronutrient fortified salt enriched with vitamin A, iron, iodine, vitamin B1, B6, B12, niacin, and folic acid. They were given enough quantity of the fortified salt to last till their next visit to the clinic which is after 3 months. The women were advised to use only the fortified salt in cooking all their meals. The women in the control group did not receive the fortified salt.

One tablet of albendazole 400 mgs in the 6^th^ month of pregnancy was given to all the women in both the groups. Deworming was carried out so that there were no worms competing for the micronutrients. This would ensure that the intestinal tract was clear for absorption of the micronutrients as in other studies [4,5].

The women in both the experimental and control groups were given iron and folic acid tablets (each tablet containing 60 mg elemental iron and 400 micrograms folic acid) which would last till their next hospital visit after 3 months. We telephoned the women periodically to ascertain whether they consumed only the tablets provided by us and found that they were consuming only the tablets provided by us.

### 2.8. Manufacture of the Fortified Salt and Dosage of the Micronutrients

A ribbon blender was used to manufacture the fortified salt. The micronutrients were added to the powdered salt and blended in a ribbon blender to get the fortified salt. After blending, samples were drawn from different places in the blender and analysed for the micronutrients of the contents. It was seen that the micronutrients were homogenously mixed with the salt after blending.

Ten grams of the fortified salt contained 2500 IU vitamin A, 30 mg of chelated iron, 30 ppm (300 *µ*g) of iodine, 1 mg of vitamin B1, 1 mg of vitamin B2, 1 mg of vitamin B6, 4 *µ*g of vitamin B12, 5 mg of niacin, and 200 *µ*g of folic acid. It can be seen that the dosage of the micronutrients is slightly less than the RDA (required daily allowance) of these micronutrients required for pregnant women. When this salt is used for cooking food, the women will get the RDA of the micronutrients. The RDA for pregnant women in India is 800 micrograms (2640 IU) vitamin A per day. Our addition of 2500 IU in 10 grams of fortified salt would ensure that the pregnant women in our study would get a dose that is slightly lesser than the RDA. The RDA of iodine in pregnant women in India is 250 *µ*g (25 ppm), but all manufacturers are mandated to add 30 ppm (300 *µ*g) of iodine during manufacture, and we also did the same.

All the vitamins and minerals added for fortifying the salt were of food grade. Vitamin A acetate, the source of vitamin A, was obtained from Max Chem Pharma, Bengaluru. Potassium iodate was the iodine source, and it was obtained from Proto Chemicals, Mumbai. Vitamins B1, B2, B6, B12, niacin, and folic acid were obtained from Crystal Pharma, Mumbai. Chelated iron was obtained from Suriksh Impex, Chennai.

### 2.9. Stability of the Fortified Salt

All the micronutrients in the fortified salt were stable for one year. The stability was tested in the laboratory at 30°C and 45% relative humidity. The details given are the mean of the 12 batches prepared for the study. We analysed the fortified salt for micronutrients on the day it was manufactured, six months after manufacture, and twelve months after manufacturing. The iodine level on the date of manufacture in 10 grams of the fortified salt was 40 ppm, and after 6 months, it was 37 ppm, and after 12 months, it was 35 ppm. The vitamin A levels on the date of manufacture in 10 grams of the. fortified salt was 3010 IU, and after 6 months, it was 2760 IU, and after 12 months, it was 2600 IU.

### 2.10. Subjects Recruited and Those Who Actually Completed the Study

There were 315 women who were enrolled in the experimental group, and 151 women completed the study. There were 334 women who were enrolled in the control group, and 150 women completed the study. It is a cultural practice in India that the woman has to go to her mother's house for the delivery of baby, especially if it is her first pregnancy. Though we asked the women before enrolment whether they would deliver in this hospital or elsewhere and even though they said they would deliver here, many of them changed their minds later. We therefore had to enroll almost double the number of women into the study since the dropout rate was very high, especially in the third trimester.

## 3. Data Collection

The women were questioned on whether they had experienced night blindness previously. They were also questioned during their visit every trimester whether they were currently experiencing any episodes of night blindness. These data were recorded.

Details about the parity of pregnancy of the women were recorded during enrolment. Details of the age of women, their occupation, and the occupation of their husbands were also recorded. Since we did not have any means to verify their salaries and arrive at their socioeconomic status, we have not analysed this aspect in this study.

In the experimental group, when the women came to the clinic during their second and third trimester, they were questioned about the number of days the fortified salt was not used in the past 3 months, and these data were recorded. This could be when they visited their relatives or travelled and ate out and hence could not consume the fortified salt provided by us.

Dietary intake questionnaire was administered once before the enrolment of the women into the study. We also collected information on the quantity of salt purchased per month by the woman as well as the number of family members in her household. The average consumption of salt being 10 grams per person per day [3], we multiplied the number of persons in the household into 10 and that value was multiplied with 30 to arrive at the quantity of salt that should have been consumed per month. For example, if the family size was 5, then 5 × 10 grams of salt per day × 30 days = 1500 grams or 1.5 kilograms of salt, which should be the salt consumption per month. We cross verified whether this was the quantity of salt that was purchased monthly by the woman. Thus, we estimated the per capita salt consumption of the women in the study. It was seen that the average consumption of salt was 10 grams per person per day. This was also seen in our previous community studies [3]. There was no difference in the dietary intake in the experimental and control groups.

### 3.1. Blood and Urine Collection and Storage

Blood was collected from the women three times during the study, i.e., in the first, second, and third trimester. Urine samples were collected twice from the women, i.e., when they were in the first trimester and in the third trimester. The tests done in the blood were for serum retinol, CRP (C-reactive protein), and AGP (alpha glycoprotein), and the test done in the urine sample was for urinary iodine concentration (UIC).

At the antenatal clinic of the hospital, after the registration process was completed, the phlebotomist collected 5 ml of blood from the women (random blood sample), and the tube with the blood was stored in a closed cupboard for a short while to enable the blood to clot. Storing the samples in a dark cupboard was to prevent light from acting and reducing the serum retinol level. The blood was allowed to clot, and serum was separated and stored in the deep freezer at minus 20°C. Urine specimens were also collected from the women. After collecting 100 urine specimens from the women in the experimental group and 100 urine specimens from the women in the control group, we stopped further collection of urine samples because from sample size calculations, we required only this number ofsamples. Urine samples were stored in the deep freezer at minus 20°C. They were periodically transported to the laboratory in Chennai on dry ice where the analysis for UIC was done.

The serum samples were transported on dry ice to the laboratory in Chennai where the analysis of serum retinol was done. The serum samples were then transported from Chennai to Germany on dry ice where the analyses of CRP and AGP were done.

### 3.2. Laboratory Analyses

Serum retinol was measured by a rapid reverse phase high performance liquid chromatography (HPLC-Shimadzu, Japan). NIST serum sample standards (lyophilized frozen serum sample SRM968c with certified retinol values) were used to calculate the retinol values. Serum retinol deficiency was defined when serum retinol was less than 20 *µ*g/dL [6]. In 10% of the samples, the serum retinol analysis was done duplicately for validation. The coefficient of variation of serum retinol was 7.2%. CRP and AGP were estimated by the Sandwich ELISA method [7], and every sample was assessed duplicately. The coefficient of variation of AGP was 4.33% and that of CRP was 10.66%. Pino et al.'s [8] modification of the Sandell–Kolthoff reaction was used to analyse urinary iodine. If the urinary iodine in the pregnant women is less than 150 mcg/L, then they have urinary iodine deficiency [9].

### 3.3. Inflammation Adjustments

The elevated acute phase proteins (CRP and AGP) in inflammation lower or reduce serum retinol values [10]. Therefore, adjustments are made if there is inflammation, and the serum retinol values are used after adjustments, so that the serum retinol values thus obtained would reflect the reality and not skewed results due to inflammation. The data were grouped according to their inflammation status [11] and adjusted serum retinol values are obtained as given in Table 1.

### 3.4. Statistical Analysis

SPSS 20.0 (SPSS Inc., Chicago IL, USA) and Microsoft Excel 2000 (Microsoft Corp., Seattle WA, USA) were used for statistical analysis. The efficacy of the intervention was analysed by comparing both the experimental and control groups. Data for serum retinol and change in serum retinol between trimesters were found to be normally distributed. Repeated measures of analysis of variance were done to compare the effects of group *x* time for serum retinol. Furthermore, we looked at changes in serum retinol between trimesters (second minus first, third minus second, and third minus first (overall)) and conducted *t* tests to evaluate these data between the groups, with Bonferroni adjustment for multiple comparison with a significant set at *p* < 0.016.

Chi-square test was done to compare the prevalence percentages of serum retinol deficiency and parity. Binary logistic regression was done to compare the effects of group *x* time for the binary variable of serum retinol deficiency with significance set at *p* < 0.05.

Since urinary iodine concentration is not normally distributed, median analysis using nonparametric tests were done. To analyze urinary iodine concentration, the Mann–Whitney test was used for the analysis of the groups and the Wilcoxon signed-rank test was used for analysing the changes within each group.

## 4. Results

### 4.1. Night Blindness

It was seen that none of the women reported any history of night blindness. Only seven women in the whole study (three in the experimental group and four in the control group) reported the prevalence of night blindness in the first, second, or third trimester.

### 4.2. Age and Parity

Details about age and parity of the women enrolled are given in Table 2.

### 4.3. Number of Days the Salt Was Not Used by Women in the Experimental Group

The average number of days the salt was not used, since the women were visiting relatives or travelling and eating out, as reported by the women in the second trimester and third trimester was 10 days and 8 days, respectively. This showed that the fortified salt was consumed regularly in the experimental group.

### 4.4. Inflammation Status

The prevalence of inflammation, CRP, AGP in the experimental and control groups in the three trimesters is given in Table 3.

### 4.5. Efficacy Study

#### 4.5.1. Serum Retinol

The serum retinol level in the three trimesters in the experimental and control groups is given in Table 4. In the first trimester, the mean serum retinol level was 24.51 *µ*g/dL in the experimental group and 28.97 *µ*g/dL in the control group, and there was a significant difference in the retinol values in the two groups. This was despite the randomization. Therefore, ANCOVA analysis was done to find out how the experimental and control groups progressed if they started at a common retinol value in the first trimester, i.e, 26.73 *µ*g/dL. ANCOVA analysis shows that in the experimental group, the serum retinol would increase to 28.04 *µ*g/dL in the second trimester, whereas in the control group, it would increase to 26.88 *µ*g/dL in the second trimester, with the experimental group faring better than the control group.

In the second trimester, in the experimental group, the retinol values increased to 27.29 *µ*g/dL, the change being an increase of 2.78 *µ*g/dL (+11.3%) when compared to the first trimester. In the control group, the retinol values decreased nonsignificantly in the second trimester to 27.63 *µ*g/dL, the change being a decrease of 1.34 *µ*g/dL(−4.6%) when compared to the first trimester.

In the third trimester, in the experimental group, the retinol values decreased nonsignificantly to 25.68 *µ*g/dL, the change being a decrease of 1.61 *µ*g/dL(−5.9%) when compared to the second trimester, whereas in the control group, there was a sharp significant decrease in the serum retinol value to 22.72 *µ*g/dL, a steep reduction of 4.91 *µ*g/dL(−17.8%) when compared to the second trimester.

If we compare the change in serum retinol between the third trimester and the first trimester, in the experimental group, there was an increase of 1.17 *µ*g/dL(+4.8%), whereas there was a decrease of 6.25 *µ*g/dL (−21.6%) in the control group.

The experimental group was significantly better than the control group in all the statistical tests.

#### 4.5.2. Prevalence of Vitamin A Deficiency

If we compare the prevalence of vitamin A deficiency defined as serum vitamin A less than 20 *µ*g/dL, in the two groups, it was seen that in the experimental group, in the first trimester, the prevalence of retinol deficiency was 39.1%, and this declined to 25.8% in the second trimester, and increased to 37.7% in the third trimester. In the control group over the pregnancy period, there was a steady increase in the prevalence of serum retinol deficiency from 14% in the first trimester to 22.7% in the second trimester to 39.3% in the third trimester. The change in the experimental group was significant (*p*=0.001) compared to the control group by binary logistic regression, as seen in Table 4.

It was seen by binary logistic regression that if the prevalence of vitamin A deficiency from only the first trimester to the second trimester is considered, it was seen that the experimental group performed significantly better than the control group (*p*=0.002). If the prevalence of vitamin A deficiency from only the second trimester to the third trimester is considered, there is no significant difference in the performance of both the experimental and control groups (*p*=0.539). If the prevalence of vitamin A deficiency from only the first trimester to the third trimester is considered, the experimental group is far significantly better than the control group, i.e., *p*=0.001. These data are given in Table 4.

#### 4.5.3. Urinary Iodine

It is seen from Table 5 that there was no significant difference in median urinary iodine values in both the experimental and control group at the baseline in the first trimester (*p*=0.064) by applying the Mann–Whitney test. This shows the homogeneity of the two groups and that both the groups are comparable. In the third trimester, by applying the Wilcoxon signed-rank test, it is found that there is a significant increase in the iodine values in the experimental group (*p*=0.030), showing the absorption of the iodine from the fortified salt. In the third trimester, there is a significant decline in the iodine values in the control group (*p*=0.008) found by using the Wilcoxon signed-rank test. In the third trimester, the iodine values in the experimental group was found to be are significantly higher than the control group (*p*=0.0001), which is found applying by the Mann–Whitney test.

## 5. Discussion

Vitamin A is extremely important for its role in vision as well as in the proper functioning of the immune system. In pregnant women, other studies have shown that as gestation progresses, the serum retinol level decreases from the first to the third trimester [12]. This is because of hemodilution where the expanding blood volume causes the decrease in the serum retinol value. The nutritional deficiency of vitamin A is also another cause for the decrease in serum retinol level from the first to the third trimester. It has been observed in other studies [13] that after delivery, the serum vitamin A increases in the mother because of the reversal in the hemodilution. In this study, we did not test the serum vitamin A in the mothers after delivery. This is one of the limitations in this study.

Pregnant women were randomized to intervention or control group by alternate week consultations of the antenatal clinic services. Hence, the randomization was not performed at the individual level. This was done for practical reasons. The antenatal clinic receives women residing in a radius of 20 kilometers around the hospital, and there is no pattern or order in which the women visit the hospital with respect to any specific geographic region from where they come. Hence, this is as good as randomization at the individual level.

In this study, we observed that in the control group, the serum retinol level decreased progressively in each trimester in the control group as the gestation progressed. However, it was seen that the experimental group received about 2500 IU of vitamin A through the fortified salt every day, and the serum retinol level increased significantly from the first to the second trimester. The vitamin A delivered through the fortified salt has helped in improving the serum retinol levels in this phase of gestation (Table 4).

As the gestation progresses from the second trimester to the third trimester, in the experimental group, there is a nonsignificant decrease in serum retinol level. This shows that the vitamin A from the fortified salt is able to help retain the serum retinol level. On the other hand, in the control group, the serum retinol value decreases significantly from the second trimester to the third trimester (Table 4).

If we consider the change in serum retinol level as the gestation progresses, at every stage of change trimester-wise, as well as the overall change over the entire gestation period, we can see from Table 4 that the experimental group is significantly far better than the control group in all the above stages. All these show that the vitamin A from the fortified salt has been able to increase serum retinol level values and maintain the levels in the experimental group, whereas the serum retinol level has decreased progressively and significantly over the gestational period in the control group.

Despite randomization, it is not clear why there is a difference in the prevalence of serum retinol deficiency in the experimental and control groups in the first trimester.

The iodine from the diet or fortified salt has to be absorbed in the body and then excreted in the urine. If there is less of iodine in the diet, the body will absorb the iodine and use it, but if there is very little iodine in the diet, then the body will absorb all of it, and the amount of iodine excreted in the urine will reduce gradually, and at one point, it will be less than 150 mcg/L, which is the cut off to define iodine deficiency in pregnant women. In pregnant women, when the urinary iodine concentration (UIC) is less than 150 mcg/L, it means they are not receiving the iodine they require from their diet. There were just 2.5% of such women in this study. 97.5% of the women in this study had sufficient urinary iodine concentrations. Still it was only the iodine from the fortified salt which caused UIC to increase in the experimental group over the study period, whereas in the control group who were consuming iodised salt, the UIC decreased significantly. The reason for this decrease in UIC in the control group is not clear. In this study, we did not analyse the iodine content in the iodised salt of the control group, which might have thrown some light on the above issue, and this is another limitation in this study. It may be possible that the need for iodine has increased in the third trimester when fetal growth is maximum, and it is the multiple micronutrient fortified salt which has been able to provide this iodine, and the iodised salt has not been able to do this, but this cannot be conclusively stated from this study because of the aforementioned limitations.

Other studies on UIC in pregnant women in North India [14] have shown that UIC data are similar to what we have obtained, so it may be presumed that in a country like India where iodisation of salt has been mandatory for the past 35 years, iodine deficiency in pregnant women may not be common.

Since vitamin A is an oil soluble vitamin and not water soluble, it cannot be easily excreted via urine, and we have ensured in this study that there are no dangers of excess vitamin A provided, by fortifying the salt in such a way that we provide less than 1 RDA(required daily allowance) of vitamin A to the pregnant women in this study.

There are some reasons for the improvement of serum retinol level in the experimental group. The fortified salt was used in all the meals cooked in the homes of the women in the experimental group. The normal eating pattern was three meals plus an evening snack, so the micronutrients were delivered in small doses throughout the day, and this may have resulted in the good absorption of the vitamin A and iodine. We have seen the improvement in serum retinol level and urinary iodine level in our previous studies with the multiple micronutrient fortified salt in children [1, 3, 15] and nonpregnant women also [16].

Thus, it can be seen that despite hemodilution, the fortified salt has been able to improve serum retinol values and maintain it at that level in the experimental group. The iodine from the fortified salt has been able to increase the urinary iodine values significantly.

## 6. Conclusion

The multiple micronutrient fortified salt given to the pregnant women in the experimental group was able to improve the serum vitamin A level and urinary iodine level in them. In the control group of pregnant women who were using iodised salt, the serum vitamin A level declined significantly, and their urinary iodine concentrations also declined significantly. Many more studies in this area need to be done for better understanding of this subject.

## Figures and Tables

**Table 1:** Inflammation adjustments done to obtain serum retinol adjusted values.

Condition	Name of the group	Serum retinol adjustments
No increase in the acute phase proteins (CRP≤5 mg/L and AGP ≤1 g/L)	Reference group	No adjustment
CRP is increased (CRP>5 mg/L) and AGP is normal (AGP≤1 g/L)	Incubation group	Serum retinol is multiplied by the correction factor 1.13
CRP and AGP are increased (CRP>5 mg/L and AGP>1 g/L)	Early convalescence group	Serum retinol is multiplied by the correction factor 1.24
CRP is normal (CRP≤5 mg/L) and AGP is raised (AGP>1 g/L)	Late convalescence group	Serum retinol is multiplied by the correction factor 1.11

**Table 2:** Parity and age of the women.

Parity:©	Experimental group (multiple fortified salt) *N* = 151	Control group *N* = 150
First pregnancy (primigravida)%	59.5	57.9
Second pregnancy%	32.9	31.1
Third, fourth, or fifth pregnancy%	7.6	11
Age (years ± SD)^#^	22.90 ± 2.73	23.0 ± 2.74

© No differences were seen in the parity between the experimental and control groups by using the chi-square test (*p*=0.347). ^#^No significant difference was seen in the age between the experimental and control groups by using Anova (*p*=0.599).

**Table 3:** Prevalence of inflammation, CRP, and AGP in the experimental and control groups in the three trimesters.

	Experimental group (multiple fortified salt) N = 151						Control group N = 150					
By trimester:	No inflammation ^*∗*^%	Incubation#%	Early convalescence§%	Late convalescence¥ %	C-reactive protein mg/L	Alpha glycoprotein g/L	No inflammation ^*∗*^ %	Incubation#%	Early convalescence§ %	Late convalescence¥ %	C-reactive protein mg/L ©	Alpha glycoprotein g/L©
First trimester	42.4	20.5	19.2	17.9	4.41 ± 3.41	0.96 ± 0.20	42	16.7	20	21.3	4.35 ± 4.54	0.97 ± 0.25
Second trimester	48.3	41.7	8	2	5.54 ± 3.80	0.76 ± 0.18	71.3	28	0.7	0	4.04 ± 3.18	0.58 ± 0.13
Third trimester	59.6	35.8	4	0.6	4.79 ± 3.58	0.63 ± 0.16	54.7	32.7	10.6	2	5.37 ± 3.62	0.74 ± 0.23

^*∗*^No inflammation is seen when there is no increase in the acute phase proteins; ^#^Incubation (when only the CRP is increased (CRP>5 mg/L) and AGP is normal); ^§^Early convalescence (when both the CRP and AGP are increased (CRP>5 mg/L and AGP>1 g/L)); ^¥^ Late convalescence (when CRP is normal and AGP is raised (AGP>1 g/L)); ©*p*-value group *x* time Anova repeat measures *p*=0.0001.

**Table 4:** Serum retinol^§^, change in serum retinol, and serum retinol deficiency^*∗*^in the three trimesters.

	Experimental group (multiple fortified salt) *N* = 151	Control group *N* = 150	*p* values
By trimester:			Repeated measures ANOVA Group *x* time *p*-value = 0.0001
First trimester	24.51 ± 10.45	28.97 ± 9.45	
Second trimester	27.29 ± 11.25	27.63 ± 10.60	
Third trimester	25.68 ± 13.07	22.72 ± 8.60	
Changes between trimesters:			*p*-values by *t*-test
Second minus first	2.78 ± 12.39	−1.34 ± 12.28	*p*-value = 0.004
Third minus second	−1.61 ± 12.82	−4.91 ± 11.59	*p*-value = 0.02
Third minus first (overall)	1.17 ± 14.19	−6.25 ± 11.15	*p*-value = 0.0001
Prevalence of serum retinol deficiency			Binary logistic regression
First trimester (%)	39.1	14.0	Group *x* time first and second trimester *p*-value = 0.002
Second trimester (%)	25.8	22.7	Group *x* time second and third trimester *p*-value = 0.539
Third trimester (%)	37.7	39.3	Group *x* time first and third trimester *p*-value = 0.001
			Group *x* time (all trimesters) *p*-value = 0.001

^§^Serum retinol, µg/dL (Mean ± SD); ^*∗*^Serum retinol deficiency is defined by serum retinol (adjusted for inflammation) < 20 *µ*g/dL.

**Table 5:** Median urinary iodine concentration (UIC)^*∗*^ mcg/L in the experimental and control groups in the first and third trimesters.

By trimesters	Experimental group (N = 100)	Control group (N = 100)	
First trimester	395 (325–474)^ac^	362 (300–417)^ad^	*p*values
Third trimester	422 (304–479)^bc^	327 (270–390)^bd^	*p*=0.0001

^*∗*^Values are median (25th–75th percentile). (a) Mann–Whitney test: No significant difference was seen between the experimental and control group at the baseline in the first trimester, so both the groups are homogenous; *p*=0.064. (b) Mann–Whitney test: A significant difference is seen between the experimental and control group at the endline in the third trimester, that the experimental group improved significantly; *p*=0.0001. (c) Wilcoxon signed-rank test: A significant improvement is seen from the first trimester to the third trimester in the experimental group, showing the uptake of iodine from the fortified salt; *p*=0.030. (d) Wilcoxon signed-rank test: A significant decline is seen from the first trimester to the third trimester in the control group; *p*=0.008.

## Data Availability

The datasets used and analysed in this study are available from the corresponding author on reasonable request.

## Abbreviations

CRP: C-reactive protein
AGP: Alpha glycoprotein
IU: International Units
Mg: Milligram
Mcg: Microgram
RDA: Required daily allowance
HPLC: High performance liquid chromatography
ELISA: Enzyme linked immunosorbent assay
NIST: National Institute of Standards and Technology, USA
SPSS: Statistical Package for Social Sciences
ANOVA: Analysis of Variance
ANCOVA: Analysis of Covariance
UIC: Urinary iodine concentration.
